# Declining Trends of Reoperations and Disease Behaviour Progression in Crohn’s Disease over Different Therapeutic Eras—A Prospective, Population-Based Study from Western Hungary between 1977–2020, Data from the Veszprem Cohort

**DOI:** 10.1093/ecco-jcc/jjad117

**Published:** 2023-07-09

**Authors:** Lorant Gonczi, Laszlo Lakatos, Petra A Golovics, Akos Ilias, Tunde Pandur, Gyula David, Zsuzsanna Erdelyi, Istvan Szita, Alex Al Khoury, Peter L Lakatos

**Affiliations:** Department of Internal Medicine and Oncology, Semmelweis University, Budapest, Hungary; Department of Gastroenterology, Ferenc Csolnoky Hospital, Veszprem, Hungary; Department of Gastroenterology, Hungarian Defence Forces Medical Centre, Budapest, Hungary; Department of Internal Medicine and Oncology, Semmelweis University, Budapest, Hungary; Department of Gastroenterology, Grof Eszterhazy Hospital, Papa, Hungary; Department of Gastroenterology, Ferenc Csolnoky Hospital, Veszprem, Hungary; Department of Gastroenterology, Ferenc Csolnoky Hospital, Veszprem, Hungary; Department of Gastroenterology, Ferenc Csolnoky Hospital, Veszprem, Hungary; Gastro Health, LLC-Care Center, Hollywood, FL, USA; Department of Internal Medicine and Oncology, Semmelweis University, Budapest, Hungary; Division of Gastroenterology, McGill University Health Center, Montreal, QC, Canada

**Keywords:** Crohn’s disease, resective surgery, biologics, immunomodulators, population-based

## Abstract

**Background and Aims:**

Few population-based studies have investigated long-term surgery rates for Crohn’s disease [CD]. Our aim was to analyse disease progression and surgery rates in a population-based cohort over different therapeutic eras, based on the time of diagnosis: cohort-*A* [1977–1995], cohort-*B* [1996–2008], and cohort-*C* [2009–2018].

**Methods:**

A total of 946 incident CD patients were analysed (male/female: 496/450; median age at diagnosis: 28 years [y]; interquartile range [IQR]: 22–40]). Patient inclusion lasted between 1977 and 2018. Immunomodulators have become widespread in Hungary since the mid-1990s and biologic therapies since 2008. Patients were followed prospectively, with both in-hospital and outpatient records reviewed regularly.

**Results:**

The probability of disease behaviour progression from inflammatory [B1] to stenosing or penetrating phenotype [B2/B3] significantly decreased (27.1 ± 5.3%/21.5 ± 2.5%/11.3 ± 2.2% in cohorts A/B/C, respectively, after 5 years; 44.3 ± 5.9%/30.6 ± 2.8%/16.1 ± 2.9% after 10 years, respectively; [*p*LogRank <0.001]). The probability of first resective surgery between cohorts A/B/C were 33.3 ± 3.8%/26.5 ± 2.1%/28.1 ± 2.4%, respectively, after 5 years; 46.1 ± 4.1%/32.6 ± 2.2%/33.0 ± 2.7% after 10 years, respectively; and 59.1 ± 4.0%/41.4 ± 2.6% [cohorts A/B] after 20 years. There was a significant decrease in first resective surgery risk between cohorts A and B [*p*log rank = 0.002]; however, no further decrease between cohorts B and C [*p*log rank = 0.665]. The cumulative probability of re-resection in cohorts A/B/C was decreasing over time (17.3 ± 4.1%/12.6 ± 2.6%/4.7 ± 2.0%, respectively, after 5 years [*p*log rank = 0.001]).

**Conclusion:**

We report a continuous decline in reoperation rates and disease behaviour progression in CD over time, with the lowest values in the biologic era. In contrast, there was no further decrease in the probability of first major resective surgery after the immunosuppressive era.

## 1. Introduction

Crohn’s disease [CD] is an idiopathic, progressive, inflammatory disorder of the gastrointestinal tract. Patients require anti-inflammatory treatment and often surgery, in order to avoid long-term bowel damage and disability.^1^ The introduction of biologic therapies—antitumour necrosis factor-α [aTNF] therapies—has dramatically changed the therapeutic landscape of inflammatory bowel disease [IBD]. Multiple randomied csontrolled trials [RCTs] have shown that aTNF therapies are effective in inducing and maintaining symptomatic remission and endoscopic healing among patients with moderate-to-severe CD.^2–4^ Novel therapeutic approaches, including early and aggressive use of immunomodulators and biologic therapies,^5,6^ aim to improve the natural disease course of CD by preventing disease progression and structural bowel damage caused by ongoing inflammation.

One of the most important disease outcomes in CD is the need for surgical interventions. Surgical resection rates varied largely over time across published studies, ranging from 25% to 61% at 5 years from diagnosis in a systematic review evaluating four decades of literature up to the early 2000s.^7^ Multiple RCTs and a meta-analysis have shown that aTNF biologics are effective in reducing hospitalisations and surgical resections.^8–10^ However, controversial results have also been published in recent years about the effectiveness of aTNF therapies in reducing surgery risk in inception cohorts. A recent, population-based, interrupted, time series study from Ontario, Canada, using health administrative databases, concluded that the marketplace introduction of infliximab was not associated with significant declines in the rates of intestinal resections or hospitalisations in CD patients.^11^ Indirect evidence is suggested from the recent Epi-IBD, population-based, inception cohort, including unselected patients from multiple European, population-based cohorts.^12^ Although significantly more patients in Western European cohorts received biologic therapy and immunomodulators, 5-year outcomes including surgery and phenotype progression were similar across Western and Eastern Europe.

To date, surgery trends in CD in the biologic era have been investigated mainly in administrative databases, and most of these are lacking extensive disease phenotype and medical management data. Very few prospective, population-based cohorts are available from the biologic era, and even fewer provide long-term data on disease course and second surgical resection rates.

The Veszprem IBD cohort is a well-established, prospective, population-based, inception cohort from Veszprem County, Hungary, originally initiated in 1977. Previous data from the same cohort concluded that the reduction in surgical rates following the late 1990s was independently associated with increased and earlier use azathioprine in this CD cohort.^13^

The present work provides the complete assessment of the Veszprem IBD cohort encompassing more than 40 years, with the aim of analysing long-term disease phenotype progression, resective surgery rates, and second surgery [re-resection] rates over different therapeutic eras, including the latest ‘biologic era’, in a prospective, population-based setting.

## 2. Methods

### 2.1. Study population and design

This study is based on a prospective, population-based, inception cohort of CD patients from Veszprem Province, Hungary. Veszprem Province is an administrative region in western Hungary, and has a population of 353 068 residents [data from national census in 2011].^14^ Patient inclusion lasted between January 1, 1977, and December 31, 2018. All patients in the investigated area who were diagnosed with CD in this period were included in the study. With each patient, diagnoses generated by in-hospital or outpatient visit records were reviewed thoroughly by an IBD expert gastroenterologist, using the Lennard-Jones^15^ and the Copenhagen Diagnostic Criteria.^16^ Time of diagnosis was defined when necessary diagnostic tests [endoscopic, histological, and/or radiological evidence] were carried out to support the diagnosis of CD. In some cases, the final diagnosis was made years after the beginning of symptoms. Case validation and supervision were performed by the lead IBD specialist gastroenterologist in Csolnoky F. County Hospital upon data centralisation. Patient follow-up ended on December 31, 2020.

The primary outcome of the study was to assess and compare long-term disease phenotype and surgery rates in CD patients between different eras of treatment paradigms. The use of immunomodulator agents [thiopurines most commonly] became widespread in Hungary from the mid-1990s,^13^ and biologic therapies have been commonly used and covered by the National Health Insurance Fund of Hungary since 2008. Therefore, we assessed the temporal trends in disease prognosis by dividing the study population into three consecutive cohorts according to the year of diagnosis: cohort A, 1977–1995 [‘pre-immunomodulator era’; cohort B, 1996–2008 [‘immunomodulator era’]; and cohort C, 2009–2018 [‘biologic era’].

### 2.2. Data collection and reporting

Patients were followed prospectively from diagnosis to the end of the follow-up period or until the date of their emigration or death. Patient data were collected from four general hospitals in the province and accompanying gastroenterology outpatient units. Patient data were collected and reviewed every year, using public health records and questionnaires filled out by treating physicians for missing/additional data. The provincial IBD register data were centralised in Veszprem. The majority of patients [over 90% of CD patients] were monitored at the Csolnoky F. County Hospital in Veszprem, with a specialised IBD gastroenterology outpatient unit that serves as a secondary referral centre for IBD patients in the province. Since 2018, a cloud-based online interface—called National eHealth Infrastructure [EESZT]—has provides access to inpatient and outpatient medical records, extensive test results, and drug prescriptions, improving the appropriateness of the data collection in our cohort. Data on disease phenotype, medications, and surgical interventions were reported and updated yearly by the treating physician, using standardised data forms. Further detailed methodological description on data collection, case ascertainment, and geographical and socioeconomic background of the province is also available in our previous publications on this inception cohort.^17,18^

Detailed demographic data were collected. Disease phenotype was evaluated at diagnosis and during follow-up based on the Montreal classification.^[19]^ Data on medical therapy were collected from patient visit medical records and prescription records, and were updated yearly. Cumulative exposures to medications were evaluated. Time to disease behaviour change, time to first biologic therapy initiation, and time to surgical outcomes were collected. Death and time to death were also registered, and follow-up times were adjusted accordingly.

This work was prepared in adherence to the STROBE guidelines.

### 2.3. Statistical analysis

Continuous variables are presented as medians with interquartile range [IQR], whereas categorical variables are presented as numbers with percentages and standard deviation [SD]. The t test or Mann–Whitney U test was used to compare continuous variables, and the χ^2^ test or Fisher’s exact test was used to compare categorical variables, as appropriate. Cumulative probabilities of medication use, disease phenotype change, and resective surgery were calculated using the Kaplan–Meier analysis, and the values were compared between groups by using the log-rank test. Multivariate regression analysis was performed to identify predictors of disease phenotype change and surgery. The following covariates were included in the statistical model: gender, age at diagnosis, era of diagnosis, disease behaviour, disease location, smoking status, and perianal manifestation. A *p*-value of <0.05 was regarded as statistically significant. Statistical analyses were performed using the SPSS software v. 20.0 [Chicago, IL, USA].

### 2.4. Ethical statement

The study was approved by the Csolnoky F. Province Hospital Institutional Committee of Science and Research Ethics [193/2004, 0712/2009, and 2/2021].

## 3. Results

### 3.1. Study population and baseline characteristics

A total of 946 patients (male/female: 496/450; mean age at diagnosis: 32.0 years[y] [SD: 14.8]) were diagnosed with CD in the inclusion period in Veszprem County. Based on time of diagnosis, n150 patients were classified into cohort A [1977–1995], 439 in cohort B [1996–2008], and 357 in cohort C [2009–2018]. Median follow-up time for the entire cohort was 15 years [IQR: 9–21]. For detailed patient characteristics and baseline disease phenotype, see Table 1. Among the three cohorts, there were subtle differences in gender distribution and age at diagnosis. In more recent cohorts, the number of male patients and mean age at diagnosis increased [*p* = 0.008, *p* = 0.003]. Decreasing rates of active smoking was observed in latter cohorts [*p* <0.001].

**Table 1.** Demographic and clinical characteristics of Crohn’s disease patients at diagnosis

	Overall cohort [*n* = 946]	Cohort A, 1977–1995 [*n* = 150]	Cohort B, 1996–2008 [*n* = 439]	Cohort C, 2009–2018 [*n* = 357]	*p*-value
Male gender	496 [52.4%]	63 [42.0%]	229 [52.2%]	204 [57.1%]	0.008
Mean age at diagnosis [SD], years	32.0 [14.8]	30.0 [11.7]	31.0 [14.2]	34.1 [16.4]	0.003
Disease location at diagnosis					0.003
* *Ileal [L1]	272 [28.8%]	43 [28.7%]	117 [26.7%]	112 [31.4%]	
Colonic [L2]	295 [31.2%]	53 [35.3%]	158 [36.0%]	84 [23.5%]	
Ileo-colonic [L3]	379 [40.1%]	54 [36.0%]	164 [37.4%]	161 [45.1%]	
Disease behaviour at diagnosis					0.004
Inflammatory [B1]	558 [59.0%]	70 [46.7%]	271 [61.7%]	217 [60.8%]	
Stenosing [B2]	177 [18.7%]	30 [20.0%]	84 [19.1%]	63 [17.6%]	
Penetrating [B3]	211 [22.3%]	50 [33.3%]	84 [19.1%]	77 [21.6%]	
Upper GI manifestation	39 [4.1%]	1 [0.1%]	10 [2.3%]	28 [7.8%]	<0.001
Perianal manifestation	165 [17.4%]	37 [24.7%]	81 [18.5%]	47 [13.2%]	0.006
Smoking					<0.001
Non-smoker	434 [45.9%]	57 [38.0%]	205 [46.7%]	172 [48.2%]	
Active smoker	409 [43.2%]	85 [56.7%]	198 [45.1%]	126 [35.3%]	
Ex-smoker	103 [10.9%]	8 [5.3%]	36 [8.2%]	59 [16.5%]	
Diagnostic delay from symptom onset to definitive diagnosis					0.013
Less than 1 year	562 [59.4%]	84 [56.0%]	250 [56.9%]	228 [63.9%]	
1**–**2 years	262 [27.7%]	36 [24.0%]	131 [29.8%]	95 [26.6%]	
More than 2 years	122 [12.9%]	30 [20.0%]	58 [13.2%]	34 [9.5%]	

SD, standard deviation; GI, gastrointestinal..

The proportion of patients with ileo-colonic [L3] disease location at diagnosis increased in cohort C [45.1%], compared with cohorts A and B [36.0% and 37.4%, respectively; *p* = 0.003]. Stenosing [B2] disease behaviour rate was stable throughout the study period, whereas penetrating [B3] disease behaviour was somewhat less prevalent at diagnosis in cohorts B and C [19.1% and 21.6%, respectively] compared with cohort A [33.3%; *p* = 0.004]. Presence of perianal manifestation at the time of diagnosis also decreased from the earlier to more recent cohorts [*p* = 0.006], whereas the rate of upper gastrointestinal [GI] manifestation at diagnosis increased [*p* <0.001]. Of note, diagnostic delay between symptom onset and definitive diagnosis was also significantly higher in earlier cohorts [*p* = 0.013].

The cumulative rates of extraintestinal manifestations over the total follow-up were as follows: hepatic manifestations: 11.5% [109/946] (primary sclerosing cholangitis: 1.5% [14/946]; non-alcoholic fatty liver disease: 8.6% [81/946]; other: 1.4% [14/946]), arthritis/arthralgia: 23.5% [222/946], ocular manifestations: 4.2% [40/946], and cutaneous manifestations: 8.1% [77/946].

### 3.2. Medical treatment

**Figure 1.**
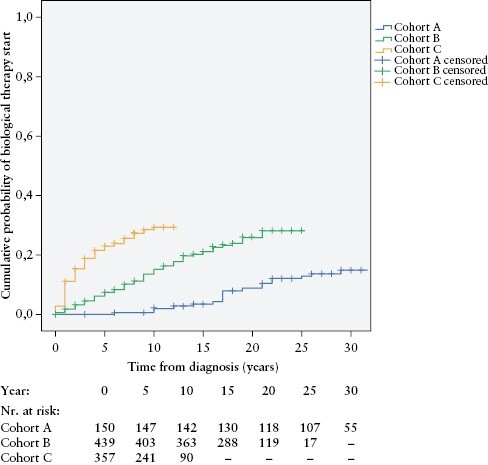
Cumulative probability of biologic therapy initiation after diagnosis in incident CD patients, stratified by time of diagnosis [cohort A, 1977**–**1995; cohort B, 1996**–**2008; cohort C, 2009**–**2018] [*p*logrank <0.001; *n *= 946].

Cumulative exposure during the total follow-up to IBD specific groups of medications is detailed in Table 2. Total 5-aminosalicylic acid [5-ASA] and corticosteroid exposure decreased over time, whereas the use of immunomodulators and biologics increased in more recent cohorts. Overall exposure to immunomodulators exceeded 60% in cohorts B and C. Overall exposure to biologics was 23.2% [219/946] in the total cohort. In all, 217/946 [22.9%] patients received aTNF biologics in the total cohort, and the cumulative rate of combination therapy with immunomodulator and an aTNF biologic agent was 19.9% [189/946] during the follow-up. Time-dependent analysis showed a 0.0 ± 0%/2.1 ± 1.2% cumulative probability for biologic therapy initiation at 5 and 10 years after diagnosis for cohort A; 7.4 ± 1.3%/15.2 ± 1.7% for cohort B; and 22.9 ± 2.3%/29.3 ± 2.7% for cohort C [*p*log rank <0.001] [Figure 1].

**Table 2.** Cumulative exposure to IBD specific groups of medications in incident Crohn’s disease patients during the total follow-up

	Overall cohort [*n* = 946]	Cohort A, 1977–1995 [*n *= 150]	Cohort B, 1996–2008 [*n* = 439]	Cohort C, 2009–2018 [*n* = 357]	*p*-value
5-ASA	824 [87.1%]	147 [98.0%]	403 [91.8%]	274 [76.8%]	<0.001
Corticosteroids	707 [74.7%]	125 [83.3%]	342 [77.9%]	240 [67.2%]	<0.001
Thiopurines	580 [61.3%]	72 [48.0%]	274 [62.4%]	234 [65.5%]	0.001
Biologicns	219 [23.2%]	21 [14.0%]	104 [23.7%]	94 [26.3%]	0.010

IBD, inflammatory bowel disease; 5-ASA, 5-aminosalicylates.

### 3.3. Disease behaviour progression

**Figure 2.**
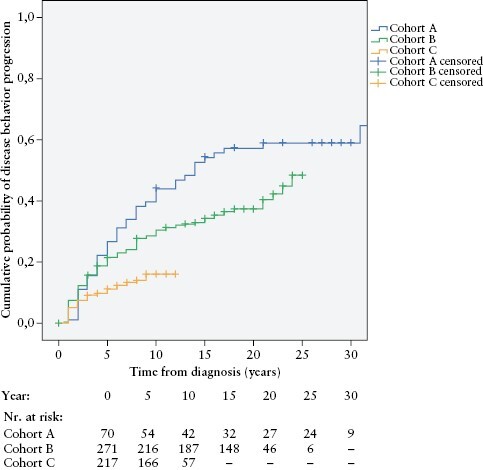
Cumulative probability of disease behaviour progression into stenosing or penetrating phenotype [B2/B3] in patients with inflammatory [B1] disease at diagnosis, stratified by time of diagnosis [cohort A, 1977**–**1995; cohort B, 1996**–**2008; cohort C, 2009**–**2018] [*p*logrank <0.001; *n *= 558].

During the total follow-up, 31.0% [178/558] of the patients with initial inflammatory [B1] disease behaviour progressed into stenosing [B2] or penetrating [B3] phenotype. A Kaplan–Meier analysis was performed to determine the risk of disease behaviour change in our CD population. The cumulative probability of disease behaviour progression in patients with inflammatory [B1] behaviour at diagnosis into stenosing or penetrating phenotype [B2/B3] was 18.4 ± 1.7% after 5 years, 27.9 ± 2.0% after 10 years, and 36.7 ± 2.5% after 20 years from diagnosis, in the total study population. Stratified by the time of diagnosis, the probability of disease behaviour progression was 27.1 ± 5.3%/21.5 ± 2.5%/11.3 ± 2.2% in cohorts A/B/C after 5 years, 44.3 ± 5.9%/30.6 ± 2.8%/16.1 ± 2.9% after 10 years, and 57.6 ± 6.0%/37.5 ± 3.1% in cohorts A/B after 20 years from diagnosis. There was a significant decrease in the cumulative probability of disease progression over time between cohorts A, B, and C [*p*log rank <0.001] [Figure 2].

A Cox regression multivariate analysis was carried out to identify predictors of disease behaviour progression. The model showed that the era of diagnosis [cohorts A/B/C; *p* <0.001], younger age [A1/A2] at diagnosis (hazard ratio [HR] 1.96; 95% CI 1.14–3.36; *p* = 0.014), ileal or ileo-colonic [L1/L3] location at diagnosis [HR 1.54; 95% CI 1.11–2.12; *p* = 0.009], perianal manifestation at diagnosis [HR 2.47; 95% CI 1.69–3.63; *p* <0.001], and smoking history [HR 1.53; 95% CI 1.12–2.09; *p* = 0.007], were predictors of progression from B1 to B2/B3 behaviour [Supplementary Table 1A].

### 3.4. First resective surgery

**Figure 3.**
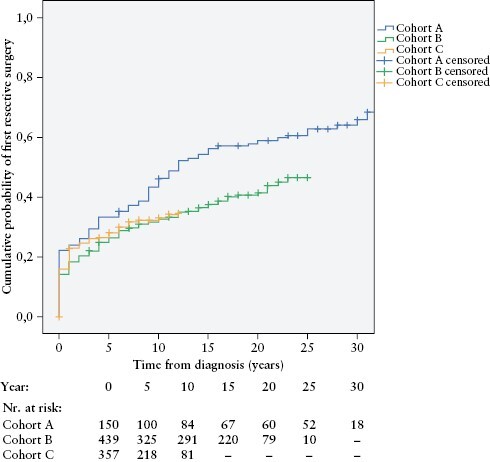
Cumulative probability of first resective surgery in incident Crohn’s disease patients, stratified by time of diagnosis [cohort A, 1977**–**1995; cohort B, 1996**–**2008; cohort C, 2009**–**2018] [*p*logrank  = 0.002; *n *= 946].

The overall resective surgery rate was 40.6% [384/946 patients] in the total cohort. The cumulative probability of resective surgery in the total study population was 28.2 ± 1.5% after 5 years, 35.4 ± 1.6% after 10 years, 45.7 ± 2.0% after 20 years. The cumulative probability of first resective surgery in cohorts A/B/C were as follows: 33.3 ± 3.8%/26.5 ± 2.1%/28.1 ± 2.4% after 5 years; 46.1 ± 4.1%/32.6 ± 2.2%/33.0 ± 2.7% after 10 years; and 59.1 ± 4.0%/41.4 ± 2.6% [cohorts A/B] after 20 years, respectively [*p*log rank = 0.002] [Figure 3]. There was a statistically plosignificant decrease in the probability of first resective surgery between cohorts A and B [*p*log rank = 0.002]. Surgery risk, however, remained similar between cohorts B and C [*p*log rank = 0.665]. Sensitivity analysis for surgery risk was performed in patients stratified by different disease phenotypes [location and behaviour] at diagnosis [Supplementary Figure 1].

A Cox regression multivariate analysis model showed that stenosing or penetrating disease [B2/B3] behaviour [HR 4.27; 95% CI 3.39–5.38; *p* <0.001] and ileal or ileo-colonic [L1/L3] location at diagnosis [HR 1.77; 95% CI 1.37–2.29; *p* <0.001] were independent predictors of first resective surgery [Supplementary Table 1B].

### 3.5. Second resective surgery

**Figure 4.**
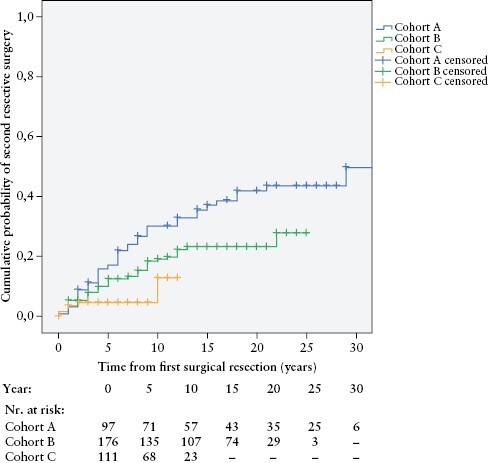
Cumulative probability of second resective surgery [re-resection] in incident Crohn’s disease patients with first surgery, stratified by time of diagnosis [cohort A, 1977**–**1995; cohort B, 1996**–**2008; cohort C, 2009**–**2018] [*p*logrank <0.001; *n *= 384].

The overall re-resection [or second resective surgery] rate among patients with a first major surgery was 20.3% [78/384] during the total follow-up. The cumulative probability of second resective surgery in the total study population was 11.7 ± 1.8% after 5 years, 20.1 ± 2.3% after 10 years, and 28.1 ± 3.0% after 20 years. After stratifying the patients based on the time of diagnosis, the cumulative probability of re-resection in cohorts A/B/C were as follows: 17.3 ± 4.1%/12.6 ± 2.6%/4.7 ± 2.0% after 5 years; 30.5 ± 5.0%/19.1 ± 3.2%/13.0 ± 5.9% after 10 years; and 42.1 ± 5.6%/23.3 ± 3.6% [cohorts A/B] after 20 years, respectively [*p*log rank = 0.001]. Figure 4. Second surgical resection risk decreased significantly alongside with more recent cohorts.

In a Cox regression multivariate analysis, only the era of diagnosis [cohorts A/B/C; *p* = 0.003] was the independent predictor of re-resection surgery [Supplementary Table 1C].

## 4. Discussion

In the present study, we evaluated data from a well-established, large, prospective, population-based, inception cohort of CD patients, extending over a period of 40 years. We assessed ‘real-life’ disease course and long-term resective surgical outcomes over three different therapeutic eras. Our results showed that there was a significant decrease in the probability of first resective surgery with the widespread introduction of immunomodulator therapies; however, surgery risk did not show further decrease in the biologic era. Disease behaviour progression rate and the probability of second surgical resection [re-resection], however, showed a continuous decrease over the different therapeutic eras, being lowest in the most recent biologic era.

Based on a systematic review of population-based studies, surgical resection rates varied widely over time in the pre-biologic era, ranging between 25–61% at 5 years, and 38–96% during the first 15 years after diagnosis.^7,20,21^ A population-based cohort from Olmsted County, Minnesota, included incident CD patients diagnosed between 1970 and 2004. The cumulative probability of first major abdominal surgery from time of diagnosis was 38.2% [95% CI 32.5%–43.5%], 47.6% [41.3%–53.3%], and 58.3% [50.6%–65.0%] at 5, 10, and 20 years, respectively. Results from the same cohort also showed that major abdominal surgery rates remained stable, with 5-year cumulative probabilities in 1970–1974 and 2000–2004 of 37.5% and 35.1%, respectively.^22^ Lakatos *et al.* published earlier results from the Veszprem IBD cohort including 506 incident CD patients diagnosed between 1977–2008. The 5-year probability of azathioprine use increased drastically, and results showed a significant reduction in the cumulative probability of resective surgery [1977–1998 vs. 1999–2008; *p*logrank = 0.022]. Early azathioprine use was significantly associated with time to intestinal resection in a multivariate Cox analysis [HR: 0.43, 95% CI 0.28–0.65].^13^Similarly, results from the present study show a notable decrease in surgery risk with cohort B, which represents the period when the use of immunomodulators increased substantially.

The Dutch South Limburg [IBDSL] population-based cohort included 1162 CD patients, grouped into three eras: 1991–1998, 1999–2005, and 2006–2011.^23^ Over time, the immunomodulator exposure increased from 30.6% to 70.8%. Similarly, biologic exposure increased from 3.1% [1991–1998] to 41.2% [2006–2011]. In parallel, the hospitaliation rate decreased from 65.9% to 44.2% and surgery rate from 42.9% to 17.4% at 5 years, [both *p* <0.01]. However, a propensity score-matched analysis was also carried out, where patients with immunosuppressive or biologic use within 2 years of diagnosis had a similar risk of hospitalisation, surgery, or disease phenotype progression compared with non-user controls [*p* >0.05 for all analyses], meaning that improvements were not significantly related to immunomodulator and biologic exposure. Authors stipulated that improvements in long-term outcomes in this cohort were mainly caused by factors other than changes in medical management, such as indications for hospitalisation and surgery, or disease monitoring.^23^ Negative results were also published by Murthy *et al*. in a population-based, interrupted, time series study from Ontario, Canada, using health administrative databases of over 20 000 CD patients between 1995 and 2012.^11^ Authors presented that prior to marketplace introduction of infliximab, there was a gradual 1.6% quarterly decline in the odds of intestinal resection. This rate of decline did not change significantly, and infliximab introduction was not associated with a statistically significant effect on intestinal resection rates [observed vs. counterfactual OR 1.10, 95% CI 0.81–1.50].

One of the most recent, and largest, population-based, multicentre cohort is the Epi-IBD inception cohort including patients from 29 European cohorts, covering a background population of almost 10 million people.^12^ The study included 488 CD patients in total, diagnosed in 2010. Comparisons were made between Western and Eastern European centres, whereby significant geographical differences were noted regarding treatment: more patients in Western Europe received biologic therapy [33%] and immunomodulators [66%] than in Eastern Europe [14% and 54%, respectively, *p* <0.01]. Cumulative probability for hospitalisations and surgery during the first 5 years from diagnosis, however, did not differ between Western and Eastern Europe [*p*logrank = 0.79 and 0.95, respectively].

Few population-based studies provide data on second resective surgery [or re-resection] rates. In the IBSEN cohort, from 1990 to 1994, 23% of the patients with first surgery required re-resection after 10 years of follow-up.^20^ The Olmsted County cohort report that the crude cumulative probability of second major abdominal surgery from time of first surgery was 30.8% [95% CI 22.6%–38.2%], 44.9% [35.0%–53.3%], and 60.8% [45.8%–72.0%], at 5, 10, and 20 years, respectively, in the total cohort [1970–2004].^22^ Second surgery risk from the Veszprem cohort is generally lower at all time points compared with data from the Olmsted county cohort, and quite similar to results from the IBSEN cohort when looking at the corresponding periods. Furthermore, we were able to assess and report time trends of the probability of second resective surgery. This is unique and to our knowledge is the first such analysis in a population-based cohort from the biologic era. We found a pronounced decrease in second resective surgery risk over time, in parallel with the introduction of immunomodulators and biologics.

Disease progression from B1 to B2/B3 phenotype slowed down significantly, with the 5-year probability decreasing from 27% to 11% in cohorts A [1977–1995] and C [2009–2018], respectively. A similar analysis was carried out in the IBDSL population-based cohort; however, authors did not observe a change in disease progression [B1>>B2/B3] over time [21.2% in the era 1991–1998 vs. 21.3% in the era 2006–2011, *p* = 0.93].^23^ In the Epi-IBD pan-European cohort, 14% of patients diagnosed with B1 disease progressed to either B2 or B3 after 5 years, with no difference observed between Western and Easter European centres [*p*logrank = 0.41]; hence in Western centres, exposure to biologics was significantly higher.^12^

Drug costs increased rapidly in the past decades with the widespread use of biologicals.^24^ The extent to which biologic agents offset these costs by preventing disease progression and reducing hospitalisations and surgery rates in the ‘real-life’ setting remains somewhat controversial. Reasons for the fact that our results also could not prove further decrease in first resective surgery rates in the biologic era may be multiple. In clinical practice, there is probably a greater variability in patient selection, therapeutic decisions, and patient follow-up than in RCTs, which may reduce treatment effectiveness at certain endpoints at the population level. Another factor may be the evolution of diagnostic tools and surgical attitudes. As is presented in our sub-analyses [Supplementary Figures 1 and 2*],* patients having stenosing or penetrating disease at diagnosis, or having developed B2/B3 phenotype during follow-up, underwent surgery somewhat earlier and at a higher rate in more recent cohorts, whereas the number of patients undergoing surgery with L1 phenotype did not decrease in recent cohorts. We did observe a progressive decrease in re-resection rates and disease behaviour progression, showing the beneficial effect of biologics. One could also stipulate that second resective surgery [re-resection] rates more accurately reflect the beneficial effect of biologics, as predominantly patients with severe disease receive biologics and most of them probably already have organ damage to some extent, which the biologics cannot reverse.

We also analysed predictive factors for surgical outcomes and disease phenotype change. Similar to our results, in the Olmsted County cohort baseline disease characteristics were significantly associated with time to major abdominal surgery: isolated terminal ileal or ileocolonic disease [HR 3.1; 95% CI 1.8–5.4], current cigarette smoking [HR 1.7; 95% CI 1.1–2.7], and penetrating disease behaviour [HR 2.8; 95% CI 1.2–6.4].^22^ In addition, colonic disease was reported to be a protective factor against resective surgery [HR 0.38; 95% CI 0.21–0.69] according to the European Collaborative Study Group [EC‐IBD] cohort [1991–1993].^25^

The strength of our study includes that the present work is one of the largest, prospective, population-based cohorts with a long-term follow-up of incident CD patients in a well-defined geographical area. This enabled us to evaluate time trends of long-term disease outcomes over different therapeutic eras. We applied strict and consistent diagnostic criteria with reliable case ascertainment, and centralised data capture with standardised data forms which were regularly updated during follow-up. Patients were consecutively enrolled and unselected. Case validation and data collection were supervised by the same lead IBD specialist gastroenterologist over the total observation period. A further advantage is the fact that our study offers the simultaneous analysis of disease phenotype evolution and second resective surgery [re-resection] rates as well, which is exceptional in current literature. Of note, we did not seek to analyse a direct association between exposure to specific therapies [aTNFs] and disease outcomes, but only to present trends on a population-based level, reflecting disease management and therapeutic algorithms in the given therapeutic era.

Limitations of our study include the subtle differences in baseline characteristics between our cohorts. Substantial increase in incidence rates has been observed in this area since 1970; hence the sharply increasing number of subjects in our cohorts.^17^ Differences in disease phenotype at diagnosis can be at least partly explained by less diagnostic delay and improved diagnostic tools in IBD over time [e.g. cross-sectional imaging].

In conclusion, this present, population-based study shows a significant decrease in disease behaviour progression and second resective surgery [re-resection] rates in the more recent therapeutic eras, showing best outcomes in the biologic era. First resective surgery rates decreased with the widespread introduction of immunomodulators; however, there was no further decrease in the era of biologic therapies. Future, population-based studies are needed to assess how current treatment strategies can influence disease progression and/or surgery outcomes in the course of CD, at the population level.

## Supplementary Material

jjad117_suppl_Supplementary_Figure_1

jjad117_suppl_Supplementary_Figure_2

jjad117_suppl_Supplementary_Table_1

## Data Availability

The data underlying this article will be shared on reasonable request to the corresponding author.
