# Supplementary figures and images for: Declining Trends of Reoperations and Disease Behaviour Progression in Crohn’s Disease over Different Therapeutic Eras—A Prospective, Population-Based Study from Western Hungary between 1977–2020, Data from the Veszprem Cohort
Source: J Crohns Colitis. 2023 Jul 9;17(12):1980–7. doi: 10.1093/ecco-jcc/jjad117 (PMC10798863; doi:10.1093/ecco-jcc/jjad117)

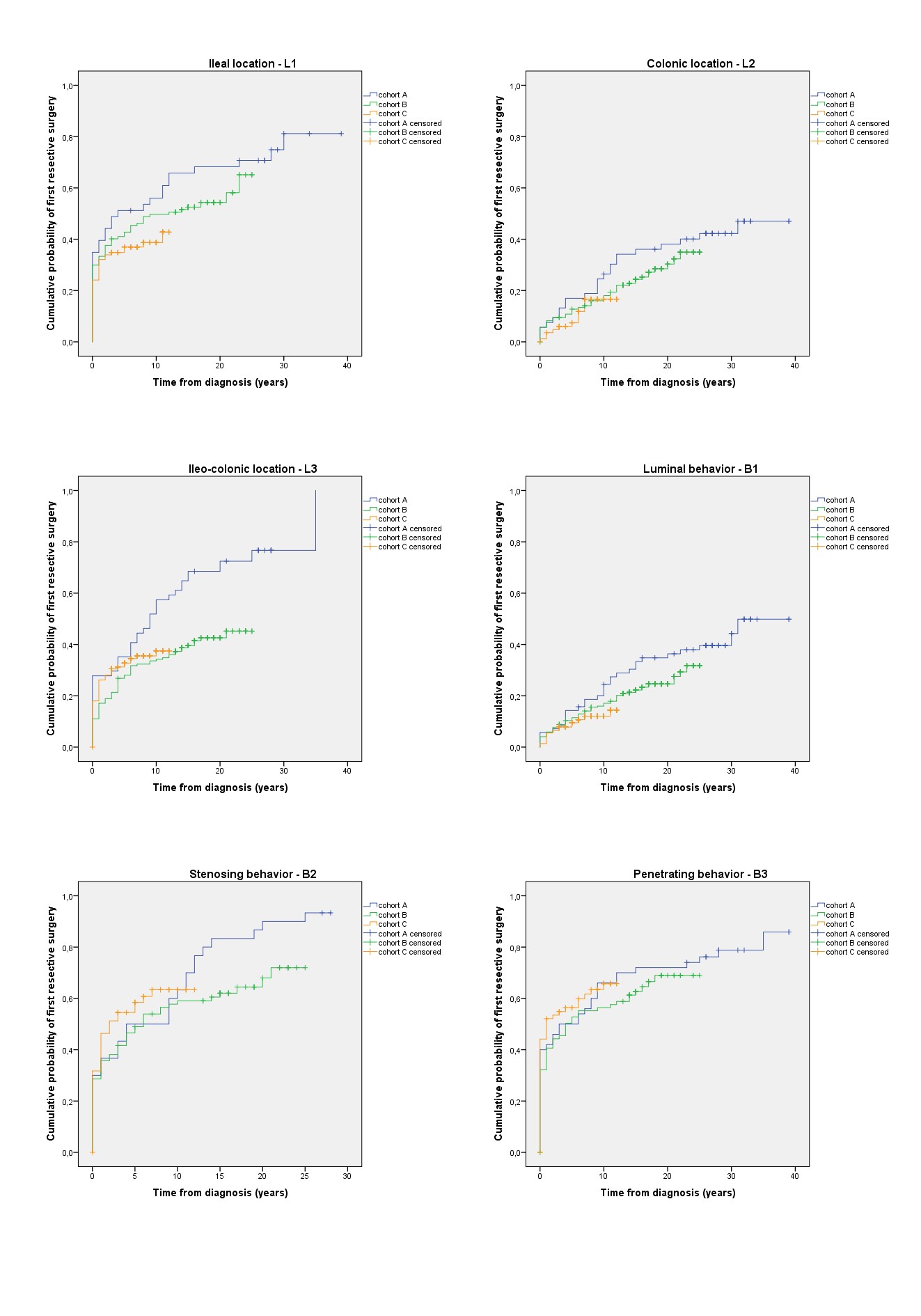

Supplement: jjad117_suppl_Supplementary_Figure_1 [file jjad117_suppl_supplementary_figure_1.jpeg]

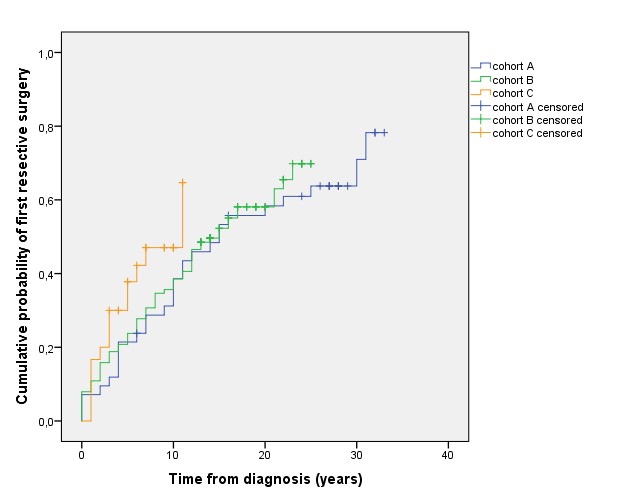

Supplement: jjad117_suppl_Supplementary_Figure_2 [file jjad117_suppl_supplementary_figure_2.jpeg]
