# Supplementary material for: Declining Trends of Reoperations and Disease Behaviour Progression in Crohn’s Disease over Different Therapeutic Eras—A Prospective, Population-Based Study from Western Hungary between 1977–2020, Data from the Veszprem Cohort
Source: J Crohns Colitis. 2023 Jul 9;17(12):1980–7. doi: 10.1093/ecco-jcc/jjad117 (PMC10798863; doi:10.1093/ecco-jcc/jjad117)
Supplement: jjad117_suppl_Supplementary_Table_1 [file jjad117_suppl_supplementary_table_1.docx]

**Supplementary Table 1. A)** Predictive factors for disease behavior progression from inflammatory (B1) to stenosing or penetrating phenotype (B2/B3) in Crohn’s disease patients diagnosed between 1977 and 2018 (n=558); **B)** Predictive factors for first resective surgery in all incident Crohn’s disease patients (n=946); **C)** Predictive factors for re-resection in Crohn’s disease patients with first surgical resection (n=384) – Cox-regression analysis

**A**

| **Factor variable** | **Multivariate HR** | **(95% CI)** | **p-valve** |
| --- | --- | --- | --- |
| Era of diagnosis (cohort A / B / C) | - | - | p<0.001 |
| Male gender | 0.92 | 0.68-1.24 | 0.575 |
| Ileal (L1) or ileo-colonic (L3) vs. colonic (L2) location at diagnosis | 1.54 | 1.11-2.12 | 0.009 |
| Perianal manifestation at diagnosis | 2.47 | 1.69-3.63 | p<0.001 |
| Smoking history | 1.53 | 1.12-2.09 | 0.007 |
| Age at diagnosis A1/A2 vs. A3 | 1.96 | 1.14-3.36 | 0.014 |

**B**

| Era of diagnosis (cohort A / B / C) | - | - | 0.226 |
| --- | --- | --- | --- |
| Male gender | 1.08 | 0.88-1.32 | 0.464 |
| Complex (B2 or B3) vs. inflammatory (B1) behavior at diagnosis | 4.27 | 3.39-5.38 | <0.001 |
| Ileal (L1) or ileo-colonic (L3) vs. colonic (L2) location at diagnosis | 1.77 | 1.37-2.29 | <0.001 |
| Perianal manifestation at diagnosis | 1.26 | 0.98-1.63 | 0.071 |
| Smoking history | 1.13 | 0.92-1.39 | 0.253 |
| Age at diagnosis A1/A2 vs. A3 | 1.05 | 0.81-1.36 | 0.708 |

**C**

| Era of diagnosis (cohort A / B / C) | - | - | 0.003 |
| --- | --- | --- | --- |
| Male gender | 1.52 | 0.96-2.42 | 0.075 |
| Complex (B2 or B3) vs. inflammatory (B1) behavior at diagnosis | 1.13 | 0.69-1.91 | 0.604 |
| Ileal (L1) or ileo-colonic (L3) vs. colonic (L2) location at diagnosis | 1.23 | 0.72-2.11 | 0.449 |
| Perianal manifestation at diagnosis | 1.13 | 0.68-1.90 | 0.637 |
| Smoking history | 1.05 | 0.67-1.64 | 0.845 |
| Age at diagnosis A1/A2 vs. A3 | 1.66 | 0.82-3.38 | 0.163 |
